# On "Spiritualism" as a Cause of Insanity

**Published:** 1863-07

**Authors:** 

**Affiliations:** Interne of the Lyon Hospitals.


### Art. X.—ON "SPIRITUALISM" AS A CAUSE OF INSANITY.

By M. Burlet, Interne of the Lyon Hospitals.*

The exaggeration of religious ideas, the belief entertained in the supernatural, the love of the mysterious, in acting vividly on the human spirit, have always been powerful causes of mental alienation.

In antiquity, in the time of prophets and magicians, subsequently, in the time of sybils and pythonesses, still later, in the days of ardent faith, of the profound piety and of the ignorance of the Middle Ages, insanity assumed a character almost exclusively religious and mystical.

The eighteenth century and its philosophy, whilst rudely assailing theocratism and ancient beliefs, until then held in the highest respect, touched but slightly or not at all on the tendencies to superstition. Then, indeed, flourished men such as Mesmer and Cagliostro, who audaciously battened upon the appetite for the marvellous.

In the present day, notwithstanding the matter-of fact and useful tendencies of scientific works the instruction which, spread throughout the masses, has enlightened and strengthened the understanding; and the scepticism which has become so general in religious and moral matters; we are almost as credulous, feeble, and silly as our ancestors were long ago, in everything which is put before us as mysterious, supernatural, or impossible. Endeavouring and affecting to believe in nothing, we accept with astounding facility things the most absurd. "Have we not been witness," says M. Morel, "of a true intellectual epidemic which has invaded us, coming from the depths of America, and which has re-animated among many persons the belief in supernatural influences? The possession of our tables and our furniture by infernal spirits, is it not a resurrection, under another form, less dangerous, happily, of beliefs which prevailed in the Middle Ages?"†

This facile credulity explains how it happens that a new theory respecting the future world, a theory pretending to unfold to us our lot and our destiny beyond the tomb, "Spiritualism" in fact (since this is the name the inventors have given to their doctrines), spreads at the present time so widely amongst us, and

* *Du Spiritisme considéré comme cause d'Aliénation Mentale.* Paris, 1863. Originally published in the *Gazette Médicale de Lyon.*

† Traité des Maladies Mentales.

must be added to those causes of insanity which are already sufficiently numerous, and which are contingent upon the manners of the epoch. What is this doctrine?

The Spiritualists profess that souls, when separated from the bodies they have animated, maintain in an invisible world a peculiar and independent life, during which they preserve the knowledge of good and evil, right and wrong, and enjoy more or less happiness, according as they have acted well or ill in their material existence. After having, for a certain length of time, been punished or recompensed for their terrestrial actions, the souls become re-incarnated in another body, and constitute in their successive transmigrations, either on the earth or on some other habitable globe, individualities and personages more or less important, according to their anterior merits. Moreover, during their existence as *spirits*, the souls can place themselves in relation with the material world.

Note in what fashion, little by little, a persevering charlatanism has succeeded in giving form and substance to a superstition which twelve years ago barely existed. Not merely by blows struck upon doors and walls, or by the dancing of tables and other moveables, do spirits now manifest themselves to us; not merely infernal spirits, as in former times, but the souls even of the dead, of our parents, our friends, and others whom we have never known, communicate with the living—write with our hands, speak with our voices, and reveal secrets, more or less important, of the invisible world, according as the spirits which bring them are more or less elevated in the hierarchy, in the social scale of the empire, and according as we ourselves are more or less worthy of these high favours. This for the moral advancement and greater happiness of the human race!

Ably worked by individuals named *mediums*, doubtless because they serve as intermediaries between the spirits and the terrestrial world, Spiritualism has made in a brief period rapid progress. The adepts, and the proselytes that the belief reckons, become more numerous from day to day. It is in towns, and in large cities especially, that Spiritualism has produced its priests, and found its principal field for culture; the agglomeration of a great number of persons has always been favourable to the development of ideas, good or bad, and to the success of charlatans. But the limit of the evil may be foreseen, for many cities have become foci from which the Spiritualist light radiates, and must sooner or later spread to its utmost, throughout the country.

The intelligent portion of the population, with few exceptions, has not failed to see in Spiritualism a gross imposture practised on a large scale. In effect, women, young girls particularly, with a good number of workmen, more or less ignorant or idle, from the immense majority of the faithful.

Lyons, for its part, has already furnished a fine contingent of madmen from Spiritualism. This city, where intellectual and other juggleries have always obtained a very happy success, has become, as it were, the stronghold of the sect. According to the avowal of a medium of Brotteaux, the number of his adherents has, within eighteen months, been prodigiously augmented. "Lyon," says M. Figuier, with its heights, the ridge of the Croix-Rousse and the summits of the Fourbières, represents admirably what the Spiritualists call a fatidical place." Thus it is not surprising that this city which, at the end of the last century, built a temple to the great thaumaturgist Cagliostro, accepts readily the celestial words with which the spirits daily favour it.

The partisans of spiritualistic ideas maintain, without proof, that their doctrine is incapable of producing mental alienation. Nay, one of them even pretends that Spiritualism is a sure preservative against insanity, and for the following reasons:—"Among the more numerous causes of undue cerebral excitement must be counted disappointments, miseries, and crossed affections . . . . now the true *spirit* sees the things of this world from a point of view so elevated, that they appear to him petty and mean, contrasted with the future which awaits him; life is for him so short and fugitive, that tribulations are but the disagreeable incidents of a voyage. That which in another person would produce a violent emotion, affects him but slightly. He knows that the chagrins of life are but trials which serve to his advancement, if he undergoes them without a murmur, because he will be recompensed according to the degree of courage with which he will have supported them. His convictions, then, give him a resignation which preserves him from despair, and, consequently, from an unceasing cause of madness and suicide." Eighteen centuries and a half ago the apostles of Christianity taught some such ideas; but the founders of our religion little expected that one day these familiar doctrines would be made use of to prop the notions of the Misses Fox.

For the rest, it is one thing to affirm and another to prove; and I look upon the portrait of the Spiritualist here given as fantastic and imaginary. My opinion is that the ideas of mediums are quite capable of troubling the mental faculties. Not that I pretend that the insanity resulting from spiritualistic practices always assumes a form related to the cause giving rise to it. No: although we do not know "to what extent the determining causes of insanity influence the forms of mental alienation" (Parchappe), it is matter of familiar observation that the delirious ideas very often have not any relation with their producing causes. Religious delirium, for example, and the delirium of persecutions, can scarcely ever be attributed to a religious sentiment carried to excess, or to persecutions endured; and it is not among persons elevated by fortune in their social position that the delirium of grandeur ordinarily manifests itself.

If, in the cases we are about to report, the majority of the subjects attacked with mental alienation suffer from a form of delirium which does not recal the cause, it does not follow that we ought to look upon Spiritualism as incapable of producing insanity; but solely that the delirium produced by this, as by any other cause, will be "in relation with the degree of the individual's sensibility, his education, manners, and character, and with the natural tendencies which render him more accessible to fear, choler, hate, jealousy, &c." (Morel.)—In relation, in a word, with the habitual passions and ideas of the lunatic.

If it be asked in what manner Spiritualism acts in the production of insanity, I answer that it does not act differently from other moral causes which may induce mental aberration. These causes, by acting suddenly or slowly upon the organ of intelligence, upon the brain, and consequently on the intellect itself; by concentrating the thoughts more frequently upon a solitary object, and isolating it from everything which does not belong to this object—these causes, I say, end by unsettling and disordering the operations of the mind, by weakening its resilience, and delirium establishes itself. This delirium will be variable, that is to say, it will have diverse forms in diverse individuals.

My object is to prove that spiritual practices act as a direct and efficient cause of insanity, and consequently, that Spiritualism ought to have a place among the causes of mental maladies. For several years the Hospice of Antiquaille, Lyon, and other special establishments of the department of the Rhone have given refuge to a great number of unfortunates, become mad from having sought for *mediumnity*. From among these I derive my observations.

CASE I.—*Chronic Mania: ambitious delirium.*

M. Th——, aged 55 years, of unknown parents, agriculturist, temperament very nervous, primary instruction passable. He is married, but without children. For thirty years he has been catarrhal, and for many years subject to dysentery in the spring. He has never had a serious illness, he is soberly religious, very temperate, and of irreproachable character. In April, 1861, this man, who up to this period had not exhibited any signs of derangement, began to occupy himself with Spiritualism and mediums, at first without attaching importance to the subject, but presently he told his wife that his hand, guided by spirits, wrote responses to questions addressed to him. If he were consulted upon the fate of persons who were dead, his hand answered in spite of himself. He replied to the cautionary observations of his wife with delirious notions. One night, notwithstanding his religious sentiments, he set himself to preach against the Archbishop of Lyon and the priests of the diocese. Placed by his wife in the establishment of Saint Georges, Bourg, he remained there three months without undergoing any amelioration; on the contrary he became covetous and gluttonous. On his return home the delirium persisted. Th—— maintained that he had two wives, and that it was his duty to teach the precepts of agriculture; he no longer recognised his friends. Having fled many times from his residence, he was at length arrested by the police and sent to Antiquaille, the 28th October, 1861. In this hospice the state of Th—— remained the same. He styled himself inspector-in-chief of agriculture, and under this title he avowed that he visited Holland and Italy, preaching there and glorifying country labours. His ideas in reality were not modified; but in addition to his passion for farming, he believed himself a priest and an angel, and he would pass the day in admiring the sun. He was perfectly calm, spoke little, and never worked. The organic functions were not disturbed. My friend and colleague, M. Chambard, who has recorded this case, could not detect any influence but Spiritualism as a cause of the insanity.

Case II.—*Acute Mania: mutilating lunacy; death.*

M. B., Italian, aged 38 years, bachelor, of a nervous sanguine temperament, and very lively disposition. No hereditary taint. This man, a hawker, visited annually the principal fairs in France. Every year, he passed two or three months with his parents at Lyon. He never returned to the city without bringing with him curious flowers and plants for his friends and neighbours, among whom he was esteemed as well-informed and talented in the cure of many maladies. Last year he often assisted at the spiritual sittings of a medium, and in the month of October he went away, somewhat exalted in mind already, and carrying with him many spiritualist works. He was not heard of again until the past July, when he was brought by the mounted police from Beaucaire to his parents at Lyon, he being then insane. In his delirium he spoke continually of spirits, of evocation, and of mediums; he wrote also phrases without much meaning, pretending that they were dictated by his familiar spirit. (In the spiritualist doctrine it is taught that each person has a spirit specially charged to look after him.) On the 27th July, having been shut up by his parents in their workshop on the ground-floor, B—— stationed himself at the window and maintained a long dispute with many spirits placed in the sun, who annoyed him. After this, he tore his dress to pieces, threw himself head-foremost against the walls, and ended by escaping from the apartment and casting himself, entirely naked, into the street. Seized, and held by several neighbours, he was taken before the commissary of police, who, however, refused to interfere. B—— then, left alone on the public road, went, still naked, to the quarter Terreaux, where the military post stopped him. An attempt to clothe him failed. He tore everything to pieces, and he had to be tied up in a sack before he could be removed to the central police office, where he was locked up in a cell. During the night he uttered loud cries, struck his head against the walls of his prison so forcibly that the blows were heard in neighbouring cells, and lacerated his visage. Dr. Serullaz, called to see the prisoner on the 28th, found him resting against the wall, entirely naked, bloody, the lower lip hanging in a shred already blackish, but suffering no pain from this great, irregular, and scarcely bleeding wound. Transported to Antiquaille, B——, on the way, fixed his eyes continually on the sun, menacing the spirits which he imagined that he saw there. On his arrival at the hospice superficial gangrene of the scalp at the summit was observed, with ecchymosis of the eyelids. The agitation and loquacity were extreme. When B—— was asked why he mutilated himself, he answered that his family were the first to place foot in Cisalpine Gaul; and that then they had made a pact with a spirit named Cêtre. This powerful spirit had wished to take possession of him, but he had resisted, and in the struggle he had received his wounds. He thought himself very fortunate in having escaped with so little harm, as the spirit might have overthrown him. B—— is an ardent medium. Often spirits speak to him, and make him write in spite of himself. He asserts that if he had not had an iron will he would certainly have become mad; that the study of spiritualism is very dangerous for the vulgar; and that the books of mediums ought to be burned. During the time that the patient was in the asylum, his delirium was general, with very great predominance of spiritualist ideas. He often sought to mutilate himself afresh to obey a spirit's order. Grave signs of mischief in the chest presently appeared, and the patient died on the 7th August of meningitis complicated with pneumonia, without having shown an instant's reason.

This case proves clearly that in certain instances the delirium may assume a form in relation with the producing cause.

CASE III.—*Acute Delirium followed by Melancholy.*

Mlle. S——, aged 29 years, mantua-maker, of Lyon. This female, of a lymphatic temperament, the catamenia at all times regular, had received good primary instruction, was very pious, irreproachable in her manners, and of a melancholy and reserved character. There was no hereditary taint. In the month of February last, Mlle. S—— accompanied a friend to a spiritual *re-union*, and vividly impressed with what she saw, she attended many sittings, and spoke of nothing but spirits, of the reality of their existence, of their power, &c. Becoming disgusted with her work, she loved to shut herself in her chamber to consult the spirits, and to write their answers and their counsels. I possess several of the written communications. The following are specimens which were often repeated: "Pray for me, my infant; the temple of God was not built in a day; have faith; I promise it to thee." Other sentences have no signification but to the writer. In the month of July, S—— became more sombre, more concentrated in herself, began to show unequivocal signs of mental alienation, and had hallucinations. She heard, in her breast, imperious voices which commanded her to rise, go out, and injure the purchasers who came to her mother's house. She pretends, moreover, that her head is entirely empty, and maintains that she is the Virgin, the mother of Jesus Christ, writes and comments on verses in the Evangel, &c. On the 22nd of August, there was an access of furious mania. Mlle. S—— threatened to kill all who approached her and to kill herself, pushed over three persons who endeavoured to restrain her, and attempted to bite when she was touched. The next morning this agitation was followed by slight prostration; but the reason not returning, the case was placed under the care of Dr. Lacour, in the Hospice of Antiquaille, and she remains there under treatment. Mlle. S—— is now calm and tranquil, does not reply when questioned, and pronounces but a few incomprehensible words in the course of the day. Her pale face expresses the gentleness of her melancholy. When she is spoken to of Spiritualism she turns away, but without anger. The organic functions are not disturbed.

CASE IV.—*Acute Delirium: death.*

M. F. R.——, farmer, aged 75 years, dwells alone in a village, in the neighbourhood of Lyon. He is still a very vigorous man, who appears always to have enjoyed good health. He was brought to Antiquaille on the 10th of December, 1861, attacked with acute, intense, and general delirium. The physician to whose care he was consigned, attributed his insanity, which had existed only three days, to "his desire to examine thoroughly the new science or religious doctrine of spirits." In the midst of incoherent loquacity the words apparition, spirits, mediums, and analogous terms, could be distinguished, but they could not be looked upon as constituting a predominant character of the delirium. The state of this patient quickly became worse; evident symptoms of meningitis supervened; and R—— succumbed on the 17th. December, three days after his admission into the asylum.

CASE V.—*Acute Mania: ambitious delirium.*

Mlle. V——, aged 43 years, of lymphatico-sanguine termperament, no children, always very regular. She had received a good primary instruction, and was very pious. Her character was gentle and peaceful; her parents died at a very advanced age; there was never a lunatic in the family. One of her uncles, M. B——, a retired, gouty captain, had served in the wars of the Empire. V—— loved to make him recount his campaigns, and to read the history of Napoleon and of great warriors. Until 1862 she lived by her work as a seamstress. Towards the end of 1861, M. B——, the captain, aged 77 years, became extremely infirm in consequence of his gouty pains and a cardiac affection. Mlle. V—— lavished her cares upon her uncle, whom she loved much, until his death, which occurred on the 6th January, 1862. Some days after the death of her husband, Madame, the widow B——, heard, or believed that she heard, during the night, and even in the daytime, violent blows struck upon the furniture, in the closets, behind the portrait of the defunct, and the doors opened and closed noisily without apparent cause. Mlle. V——, when her aunt told her of these strange noises, was vividly impressed. In the end, Mme. B—— consulted a medium, and through him the spirit of M. B—— wrote a letter, in which he implored prayers and a nine days' devotion, related his sufferings, and finished by asking for a clock, for time hung heavily in Purgatory where he still remained. The clock demanded was entrusted to the medium to be sent to its destination! Mlle. V—— kept the letter a week, and commented upon and thought of it continually. Suddenly she became gloomy, began to speak of the dead, of the devil, spirits, &c. In February she was seized with acute mania, and during the attack she broke or injured whatever she could lay her hands upon, tore her clothing and the curtains, sung songs of war and in honour of Napoleon, declared that she wished to visit the court to attend upon the Empress, &c. In this condition she was brought to Antiquaille on the 18th February, 1862. For fifteen days about, the same state continued; sometimes, indeed, it was necessary to use a strait-waistcoat and fasten the patient to a restraint-chair. Gradually she became calmer, and an interval of three months occurred, during which, if V—— was not entirely lucid, she at least, up to a certain point, was conscious of the nature of her acts. In the middle of July a fresh paroxysm of mania supervened, characterized by the same phenomena and very acute. The delirium was general, with predominance of grandiose ideas. V—— sung war songs, spoke incessantly of Napoleon, whom she wished to invite with the other crowned heads of Europe to dinner, was about to marry an emperor, &c. The agitation was extreme, and the strait-waistcoat and chair were necessary. At the end of three weeks the delirium abated, reason returned, and now Mlle. V—— is fully convalescent.

In this case frequenting mediums has not been necessary to produce insanity. Simply reading a letter written under presumed spiritual influence has sufficed to bring about the sad result.

CASE VI.—*Acute Mania: religious delirium.*

F. X——, aged 42 years, of a nervous bilious temperament, and somewhat unsociable character, well instructed, having no religious sentiment, and a great smoker. There has been no lunatic in his family, and he himself has always been well. He was received into Antiquaille the 5th September, 1861. X—— dwelt in the most distant part of the Brotteaux, that is to say, in a part of the city of Lyon where mediums are most in vogue, and where nearly every one is, more or less, a spiritualist. Having heard speak of spirits and of their doings, X—— obtained the classical works on the doctrine of mediumnity, and he read them with fervour. He presently became himself *a writing and seeing medium.* Towards the end of June, 1861, he became singular in his acts and gestures; then he had hallucinations of sight and hearing. From time to time, and whilst he worked at his loom, a brilliant light would strike his eyes, and brighten his piece of silk, then all would become obscure. For two months he saw constantly by his side, or before him, his familiar spirit. This accompanied him everywhere, at table and at work; and when X—— was walking, it would indicate to him the places he ought to evade. This spirit had the form of a man of beautiful stature, with long curling hair, falling upon the shoulders, and (a peculiarity to be remarked) the part of the body situated between the navel and the middle third of the thigh was aëriform, did not exist. At other times, X—— heard voices which gave him orders in a very imperious tone. If he did not obey immediately, he felt himself pricked by needles over his whole body; if, on the contrary, he was docile, the voices promised to restore him the sight of his youthful days. The delirium of X—— was general, but notwithstanding religious ideas were very dominant, although he was far from being pious. He declared that as the result of an invocation made by him, St. Cecilia, the patroness of musicians (X—— played passably on the violin), had appeared to him. He confounded Jesus Christ and the Virgin with the Emperor and Empress. X—— spoke slowly but much, and often he became inextricably involved in his phrases. He imagined that he saw God, and that God spoke constantly to him; every moment he conversed with an invisible interlocutor. Before answering questions put to him, he prayed to God to come to his aid and inspire him with wisdom, &c. Here is a specimen of his most habitual responses:—" I am a great prophet, a great prophet whom God has sent into a lunatic asylum in order to preach in seven languages the Catholic religion, apostolic and Roman, the Protestant religion, the Mohammedan religion: yes, I know I speak seven languages." At the end of a month of treatment under the direction of Dr. Arthaud, M. X—— was able to return home completely cured. I saw him a few days ago. His health was perfect; he meddled no more with spiritualists; his reason was intact, and he now superintends an important manufactory of tulles.

In all the preceding cases I hold that the mental alienation was certainly occasioned by Spiritualism.

When, in fact, we see so many individuals of different ages, manners, education, character, and temperament, in whom it is impossible to trace the influence of the most powerful cause of all in the genesis of insanity, that is to say, hereditage; when we see these individuals all arrive at the same result, insanity, after having all abandoned themselves to the same error, Spiritualism, it is impossible that there should be in this fact but a simple coincidence. Here, evidently, the same cause has produced the same effects.

And if it be objected that this cause gives rise to different forms of delirium, I repeat that which I have already said at the commencement of this paper; to wit, that the nature of the delirium depends not on the cause which produces it, but upon the moral and physical condition of the delirious person.

It is thus that in the cases cited, the forms of delirium are variable as the subjects themselves.

The same holds good not only for insanity, but also for all other pathological facts. Does not clinical observation prove to us daily that one and the same cause acting upon a great number of individuals, may induce in each of them a like morbid state, modified in its forms according to the diversity of constitutions, temperaments, and habits of the subjects attacked?

These cases are not all that could be brought forward to prove the danger of Spiritualism. Other special establishments of the department have received a good number of lunatics whose lunacy admitted of no other explanation than frequenting mediums. Dr. Carrier for his part, and within a short space of time, has treated and seen recover among his patients three females who had been rendered insane by Spiritualism; and I believe that there are few physicians who, of late years, have not met with similar cases.

I would reply here to a remark which might be made, that I have only met with spiritualistic lunatics among the more humble classes of society. This is true, because those patients received at Antiquaille, in the immense majority of cases, if not always, are the poor and indigent. But, independently of the names cited towards the close of this paper, I know from trustworthy sources, that the gates of a well-known *maison de santé*, principally resorted to by the rich, have admitted within its walls victims of Spiritualism from among the aristocracy.

It may be said that, having regard to the number of those who study and practise intercourse with mediums, the number of lunatics is very restricted. This opinion is not well grounded; the lunatics from spiritualism are not solely those whom it is found necessary to confine in an asylum. There are many, and of these I know many, who, although they have not reached the same condition as those whose histories I have related, still give proofs daily of being more or less stricken in their intellectual faculties. In this respect the common sense of the public has long outstripped science.

In the second place, Spiritualism, had it but produced a single case of insanity, this solitary disaster, it appears to me, would not be compensated by all the advantages which the mediums promise.

This is not the first time that the influence of Spiritualism in fostering intellectual disorders among those who cultivate the belief has been pointed out. In America, the country which gave birth to this delusion, and where it is in great favour, the number of cases of mental alienation occasioned by it is prodigious. A United States' journal declared in 1852—"The majority of the mediums become haggard, idiots, mad or stupid; and it is the same with many of their auditors. Not a week passes in which we do not hear that some of these unfortunates destroy themselves by suicide, or are removed to a lunatic asylum. The mediums often manifest signs of an abnormal condition of their mental faculties, and among certain of them are found unequivocal indications of a true demoniacal possession. The evil spreads rapidly, and it will produce in a few years frightful results." *

In France, have not individuals belonging to the elevated classes of society also been victims of the destructive power of Spiritualism? An advocate of Paris, Victor Hennequin, who placed himself in relation with the soul of the earth by means of tables; and who, under the influence of Spiritualism, wrote the opuscule entitled "*Sauvons le genre humain*," died in a lunatic asylum, after having placed his wife, who became lunatic from the same cause, in another asylum. A distinguished man of science, Girard de Caudemberg, a civil engineer, died also lunatic, in 1858, after having published a spiritualistic book, entitled "*Le monde spirituel.*"

Among all classes of society Spiritualism has found adepts and victims, and unhappily the prediction of the journal just quoted has been fully realized.

It is fitting now to state the influences and causes to which the success of this belief is to be attributed.

I do not say with the author of the book of spirits that Spiritualism has spread so quickly because it "alleviates the bitterness of life's chagrins, calms the despair and agitation of the soul, dissipates the uncertainties or the terrors of the future, arrests the thought of shortening life by suicide," &c. These assertions require to be proved, which would not be easy. No; the sources of this lamentable progress must be sought elsewhere.

The causes of the propagation of Spiritualism are the same, modified by the manners and knowledge of our time, as those under the influence of which grew and were propagated in former ages many analogous intellectual epidemics, such as the demonolatry in Lombardy in 1504, in Lorraine in 1580, in the Jura in 1598, in Spain in 1630; and the vampirism in Poland, Hungary, and Moravia from 1700 to 1740. Scarcely three years have elapsed (1859) since an epidemic of hystero-demonomania was observed at Morzine (Haute-Savoie) as reported in the *Gazette Medicale de Lyon*, by Dr. Arthaud. (†)

---

* *Boston Pilot*, 1st June, 1852. Quoted by M. L. Figuier, in his *Histoire du Marveilleux*.

† See a brief account of this epidemic, p. 189 of the present volume of the *Medical Critic*.

These causes rest often "in that taste for the marvellous so anciently and universally diffused; and in the fact of the progress of superstitions being as much more rapid as they have proved to be most absurd."*

"This love of the marvellous," says M. Figuier, "is not peculiar to our epoch; it is of all times, and of every country, because it appertains to the human mind. By an instinctive and unjust distrust of his own powers, man is led to place above himself invisible powers, exercising themselves in an inaccessible sphere. This natural disposition has existed at every period of the history of humanity, and invested, according to the period, the place, and the manners, with different aspects, it has given birth to manifestations variable in their form, but at the bottom identical in principle."

If, then, man is naturally inclined to have religious beliefs, ancient doctrines being greatly enfeebled in consequence of the attacks of which they have been the object, and of the ridicule cast upon them, the birth of a multitude of religious essays, more or less ingenious, is explained and justified. It is on this account that, after many doctrines have disappeared, Spiritualism has been developed.

To these general causes let me add, and of Spiritualism in particular, that there is something very seductive for sensitive, and an allurement for eager souls: for the first the almost certitude which Spiritualism proffers of knowing the lot of those whom we have loved and lost; for the others, the hope of learning things relative to and important to their material interests, because the spirits ought naturally to know all things, and any question may be addressed to them. Moreover, let us not forget the ability which the authors of the sect have displayed in not attacking or meddling with anything in politics or religion. The latter even renders them very useful help. For, taught by our religious books, Catholics, Protestants, and other members of the great Christian family, must alike repose faith in the celesto-corporeal apparitions of saints, of angels, of God himself, and of other prodigies even more surprising. Are not our understandings, then, as soil, well prepared for receiving and causing to germinate and fructify the seed sown by mediums? But I hasten to add, in order that no one may misapprehend me: "It is not religion, but superstition, which engenders insanity."†

If, in other parts of France, cases of insanity induced by the doctrines of the mediums, are as frequent as in the department

---

* Fodéré, *Traité du delire.*

† Parchappe.

I dwell in, no reason to the contrary existing, it seems to me that there can be no doubt that Spiritualism should rank among the most fruitful causes of mental alienation.

If we are pained to witness so much mischief, produced by a cause so foolish, we are reassured and consoled by the reflection, that as Spiritualism does not rest upon a sound basis, it will never become universal and durable. Sooner or later it will be reduced to the condition of a sect having but the blind pride of its adepts to sustain it. Common sense will not fail to resume its rights, and do justice. Spiritualism will disappear as ancient superstitions have disappeared, of which, indeed, it is a continuation under a different form—to be succeeded, doubtless, by some new error.

But, be this as it may, we cannot at the present time be too careful in setting bounds to the speculations of mediums and impeding their progress; in this way diminishing their fatal influence. To this end, would it be well to demand the intervention of public authority? No: let us not give to Spiritualism the prestige of a persecution which would but augment its success.

It is for science, and principally for medical science, and healthy religious doctrine, to combat this absurd American innovation, since it has assumed among us the dangerous importance of an epidemic, and almost the authority of a dogma.

Moreover, the heads of families and of workshops, &c. should caution their children or their workpeople against frequenting those spiritualist reunions called *circles*, in which danger to the reason is not the sole harm to be feared.

